# Specialist laboratory networks as preparedness and response tool - the Emerging Viral Diseases-Expert Laboratory Network and the Chikungunya outbreak, Thailand, 2019

**DOI:** 10.2807/1560-7917.ES.2020.25.13.1900438

**Published:** 2020-04-02

**Authors:** Giulietta Venturi, Stephan W Aberle, Tatjana Avšič-Županc, Luisa Barzon, Christoph Batejat, Elisa Burdino, Fabrizio Carletti, Rémi Charrel, Iva Christova, Jeff Connell, Victor Max Corman, Mary Emmanouil, Anne J Jääskeläinen, Ivan Kurolt, Yaniv Lustig, Miguel J Martinez, Marion Koopmans, Orsolya Nagy, Trung Nguyen, Anna Papa, Mercedes Pérez-Ruiz, Martin Pfeffer, Jelena Protic, Johan Reimerink, Giada Rossini, María Paz Sánchez-Seco Fariñas, Jonas Schmidt-Chanasit, Sandra Söderholm, Bertrand Sudre, Marjan Van Esbroeck, CHIKV-Working Group, Chantal B Reusken

**Affiliations:** 1 Department of Infectious Diseases, Istituto Superiore di Sanità, Rome, Italy; 2 Center for Virology, Medical University of Vienna, Vienna, Austria; 3 Institute of Microbiology and Immunology, Faculty of Medicine, Ljubljana, Slovenia; 4 Department of Molecular Medicine, University of Padova, Padova, Italy; 5 Laboratory for Urgent Response to Biological Threats (CIBU), Institut Pasteur, Paris, France; 6 Laboratory of Microbiology and Virology, Amedeo di Savoia Hospital, Turin, Italy.; 7 National Institute for Infectious Diseases 'Lazzaro Spallanzani' IRCCS, Rome, Italy; 8 Unité des Virus Emergents (UVE: Aix Marseille Univ, IRD 190, INSERM 1207, IRBA, IHU Méditerranée Infection), Marseille, France; 9 National Reference Vector-borne pathogens Laboratory, National Center of Infectious and Parasitic Diseases, Sofia, Bulgaria; 10 National Virus Reference Laboratory, University College Dublin, Ireland; 11 Department of Virology, Labor Berlin, Charité-Vivantes GmbH, Berlin, Germany; 12 Charité-Universitätsmedizin Berlin, corporate member of Freie Universität Berlin, Humboldt-Universität zu Berlin, and Berlin Institute of Health, Institute of Virology, Berlin, Germany; 13 Diagnostic Services Laboratory, Public Health Laboratories, Hellenic Pasteur Institute, Athens, Greece; 14 Virology and Immunology, University of Helsinki and Helsinki University Hospital, Helsinki, Finland; 15 Research unit, University Hospital for Infectious Diseases ‘Dr. Fran Mihaljević’, Zagreb, Croatia; 16 Central Virology Laboratory, Ministry of Health, Chaim Sheba Medical Center, Tel-Hashomer, Israel; 17 ISGlobal, Hospital Clínic - Universitat de Barcelona, Barcelona, Spain.; 18 Department of Viroscience, Erasmus Medical Centre, Rotterdam, the Netherlands; 19 Department of Virology, National Public Health Center, Budapest, Hungary; 20 Département de Microbiologie, Laboratoire national de santé, Luxemburg; 21 Department of Microbiology, Medical School, Aristotle University of Thessaloniki, Thessaloniki, Greece; 22 Servicio de Microbiología, Hospital Universitario Virgen de las Nieves, Instituto de Investigación Biosanitaria, Granada, Spain; 23 Institute of Animal Hygiene and Veterinary Public Health, Leipzig, Germany; 24 National Reference Laboratory for ARBO viruses and haemorrhagic fever, Belgrade, Serbia; 25 Centre for Infectious Disease Control, National Institute for Public Health and the Environment, Bilthoven, the Netherlands; 26 Regional Reference Centre for Microbiological Emergencies (CRREM), Unit of Clinical Microbiology, St Orsola Malpighi Hospital, Bologna, Italy.; 27 Centro Nacional de Microbiología. Instituto de Salud Carlos III, Madrid, España; 28 WHO Collaborating Centre for Arbovirus and Haemorrhagic Fever Reference and Research, Bernhard Nocht Institute for Tropical Medicine, Hamburg, Germany; 29 Department of Microbiology, The Public Health Agency of Sweden, Solna, Sweden; 30 European Centre for Disease Prevention and Control, Solna, Sweden; 31 Department of Clinical Sciences, Institute of Tropical Medicine, Antwerp, Belgium; 32 The members of the CHIKV-Working Group are listed at the end of the article

**Keywords:** lab surveillance, chikungunya, travel, lab preparedness and response, EVD-LabNet, Thailand

## Abstract

We illustrate the potential for specialist laboratory networks to be used as preparedness and response tool through rapid collection and sharing of data. Here, the Emerging Viral Diseases-Expert Laboratory Network (EVD-LabNet) and a laboratory assessment of chikungunya virus (CHIKV) in returning European travellers related to an ongoing outbreak in Thailand was used for this purpose. EVD-LabNet rapidly collected data on laboratory requests, diagnosed CHIKV imported cases and sequences generated, and shared among its members and with the European Centre for Disease Prevention and Control. Data across the network showed an increase in CHIKV imported cases during 1 October 2018–30 April 2019 vs the same period in 2018 (172 vs 50), particularly an increase in cases known to be related to travel to Thailand (72 vs 1). Moreover, EVD-LabNet showed that strains were imported from Thailand that cluster with strains of the ECSA-IOL E1 A226 variant emerging in Pakistan in 2016 and involved in the 2017 outbreaks in Italy. CHIKV diagnostic requests increased by 23.6% between the two periods. The impact of using EVD-LabNet or similar networks as preparedness and response tool could be improved by standardisation of the collection, quality and mining of data in routine laboratory management systems.

## Background

The rapid increase in chikungunya virus (CHIKV) cases in Thailand since October 2018 raised concerns in Europe about the potential increased risk for public health of CHIKV importation through returning travellers [1,2]. Thailand is a popular tourist destination with 6.5 million European travellers in 2017 alone [3]. In the last 3 months of 2018, Thailand reported 3,314 probable and confirmed cases and in 2019, as at 27 May 2019, 3,592 CHIKV cases were reported in 23 provinces [4]. Cases of CHIKV infection imported from Thailand into Europe and the Middle East were reported in early 2019 [5,6].

Chikungunya is characterised by a rapid onset of high fever, rash and joint pain. CHIKV is an alphavirus that is transmitted to humans by the bite of some *Aedes* mosquitoes; *Ae. albopictus*, an important mosquito vector for CHIKV, is established in 15 European countries, mainly in the south of the continent [7]. Its presence has resulted in autochthonous transmission foci initiated by returning viraemic travellers in Italy and France on several occasions in the past [8-13]. It has been suggested that the A226V mutation in the E1 gene of the CHIKV genome could potentially influence the fitness of CHIKV East-Central-South African (ECSA)-Indian Ocean lineage (IOL) strains for certain *Ae. albopictus* populations [14]. This variant caused the first European outbreak in Italy in 2007 [11] and several autochthonous infection foci in France in 2014 and 2017 [8,9,12]. However, in 2016 a sub-cluster of ECSA-IOL strains without the A226V mutation emerged in Pakistan and neighbouring countries [15,16]. This E1 A226 variant caused two outbreaks with hundreds of cases in 2017 in Italy [10,13] and experimental studies showed that the Pakistani variant was as efficiently transmitted by Italian *Ae. albopictus* mosquitoes as the E1 A226V 2007 outbreak strain [17,18].

In southern Europe the *Ae. albopictus* activity season typically lasts between June and October. Its start heightens awareness regarding the risk of local CHIKV transmission following the introduction of the virus by travel-associated viraemic cases. Hence, questions arose in March 2019 with European public health authorities about the threat of the CHIKV outbreak in Thailand to Europe. An assessment of such threat would be informed by the number of diagnostic requests and imported cases as well as the CHIKV strain currently circulating in Thailand. Here, we illustrate the potential of specialist laboratory networks as preparedness and response tool by rapid collecting and sharing of data and laboratory-diagnosed CHIKV patients related to the outbreak in Thailand.

## Emerging Viral Diseases-Expert Laboratory Network

International networking is essential for preparedness and response to emerging infectious disease events. Accurate laboratory diagnosis of emerging pathogens like CHIKV may be challenging, which can be problematic as it forms the basis for adequate surveillance, clinical and public health responses and monitoring of the effectiveness of prevention and intervention efforts [19]. These notions were recognised and in response, the European Centre for Disease Prevention and Control (ECDC) funded EVD-LabNet (https://www.evd-labnet.eu/). EVD-LabNet represents a laboratory and knowledge platform for support of clinical and public health preparedness and response through laboratory capacity and capability building. Moreover it provides access for ECDC, national public health authorities and its member laboratories to reference diagnostics, expertise and knowledge exchange [20,21]. EVD-LabNet comprises 61 expert laboratories in 30 European Union and European Economic Area (EU/EEA) countries, seven laboratories in seven EU pre-accession countries, three in two other European countries and six in six non-European countries. Among the EVD-LabNet laboratories, 51 have the capacity to diagnose CHIKV infection by molecular tests and 44 by serological tests. Moreover, 22 and 29 laboratories have indicated to have the capability to isolate the virus and to characterise it through full genome sequencing and/or sequencing of PCR amplicons respectively. Sequence information is of importance for risk assessment and for the optimisation and evaluation of available diagnostic tests.

## Chikungunya cases imported from Thailand into Europe and Israel

To substantiate the need for a heightened awareness for a possible increased public health risk of import of CHIKV cases from Thailand into Europe, we investigated whether the outbreak was reflected in the diagnostic requests and outcomes at EVD-LabNet member laboratories. Furthermore, we investigated whether genetic information, in particular about the E1 gene, on circulating strains was available. On 27 March 2019, the 51 EVD-LabNet laboratories that offer molecular CHIKV diagnostics were initially asked by email whether they had diagnosed any CHIKV cases related to the then ongoing outbreak in Thailand and if sequence information was (or could) become available.

In a follow up email on 2 May 2019, responding laboratories were asked for more details about the CHIKV diagnostic data collected between 1 October 2018 and 30 April 2019 relative to the same period from 2017 to 2018. In particular, laboratories were asked to share available information about CHIKV E1 sequences obtained from viraemic patients. These data are shown in Table 1. They were used to further inform the assessment of risk of local transmission by *Ae. albopictus*.

**Table 1:** Chikungunya virus diagnostic requests and results, EVD-LabNet laboratories, 1 October 2017–30 April 2018 and 1 October 2018–30 April 2019

Reference Laboratories, country (town)	1 October 2017–30 April 2018		1 October 2018–30 April 2019			
	Number of patients		Number of patients		Sequences from CHIKV infected patients related to Thailand outbreak (no., sequence)	If yes: presence of the E1 A226V mutation
	Positive for CHIKV /total tested^a^	Positive for CHIKV/total tested^a^ known to be from Thailand	Positives/ total tested^a^ for CHIKV^a^	Positives/total tested^a^ known to be from Thailand		
Austria (Vienna)	1/199	0/19	9/266	8/26 (5 from Krabi, 2 from Ko Samui, 1 unknown)	Y (5 patients, E1 partial sequence)	N
Belgium ( Antwerp)	1/724	0/22	12/751	4/30	N	NA
Bulgaria (Sofia)	0/0	NA	0/0	NA	NA	NA
Croatia (Zagreb)	0/1	0/0	0/1	0/0	NA	NA
Finland (Helsinki)	1/101	0/15	10/64	10/24	Y (2 patients, full genome)	N
France (Marseille)	0/240	0/unknown	10/1236	1/42	N	NA
France (Paris)	0/0	NA	0/0	0	N	NA
Germany (Berlin)	2/43	Unknown	0/17	0/unknown	NA	NA
Germany (Hamburg)	18/1,283	Unknown	51/1263	1/unknown	N	NA
Germany (Leipzig)	0/0	NA	0/0	NA	NA	NA
Greece (Thessaloniki)	0/2	0/0	0/0	NA	NA	NA
Greece (Athens)	0/3	0/0	0/2	0/0	NA	NA
Hungary (Budapest)	2/146	0/32	2/120	0/29	NA	NA
Ireland (Dublin)	0/45	Unknown	2/48	1/unknown	N	NA
Italy (Bologna)	2/107	0/4	4/75	4/7	Y (1 patient, E1 partial sequence)	N
Italy (Rome, INMI)	0/170 ^b^	0/15	2/135	1/12	Y (1 patient, E1 partial sequence)	N
Italy (Rome, ISS)	1/42^b^	0/2	3/39	1/5	N	NA
Italy-(Padua)	2/165	0/8	3/137	1/16	N	NA
Italy (Turin)	0/43	0/3	1/46	1/7	N	NA
Luxembourg (Luxembourg)	0/81	0/4	0/92	0/9	NA	NA
Serbia (Belgrade)	1/1	0/0	0/0	0/0	NA	NA
Slovenia (Ljubljana)	1/20	Unknown	1/26	1/1	Y (1 patient, full genome)	N
Spain (Barcelona)	0/273 2^c^	0/unknown	0/287 1^c^	0/unknown 1^c^	Y (1 patient^c^, E1 partial sequence)	N
Spain (Granada)	2/43	1/13	0/31	0/5	NA	NA
Spain (Madrid)	8/375	0/6	2/492	0/8	NA	NA
Sweden (Solna)	3/94	0/unknown	48/514	32/unknown	N	NA
The Netherlands (Bilthoven)	1/237	0/6	2/218	1/8	N	NA
The Netherlands (Rotterdam)	1/175	0/3	5/218	4/13	N	NA
Israel (Tel Aviv)	3/85	Unknown	6/75	1/unknown	Y (1 patient, E1 partial sequence)	N
**Total**	**50/4,698**	**1/152**	**173/6,153**	**72/242**		

CHIKV: chikungunya virus; EVD-LabNet: Emerging Viral Diseases-Expert Laboratory Network; N: no; NA: not applicable; Y: yes.

^a^ Includes molecular and/or serology requests.

^b^ Only travellers from endemic regions different from Italy in 2017.

^c^ Cases diagnosed in September (outside the period of study).

Within 2 days, 39 laboratories (representing 21 EU/EEA countries, two EU pre-accession countries, two other European countries and one non-European country) responded to the initial request indicating whether or not they had diagnosed CHIKV cases and obtained associated sequences related to the increased numbers of CHIKV cases reported in Thailand. Of 39 respondents, 17 (in 12 EU/EEA, one other European and one non-European country) indicated to have diagnosed CHIKV cases related to the outbreak in Thailand. Of 39 respondents, 29 laboratories in 18 countries (17 EU/EEA and one non-European) answered to the more detailed second request for information; three laboratories (in three EU/EEA countries) had no CHIKV requests in either of the specified time periods.

Between 1 October 2018 and 30 April 2019, 6,153 patients were tested by 26 laboratories for CHIKV of which 173 (2.8%) were indicated to be probable and confirmed cases of CHIKV infection based on national case definitions. Among 242 patients with known travel history to Thailand, 72 (29.8%) were diagnosed as probable and confirmed CHIKV cases. A 23.6% increase in the total number of patients tested for CHIKV (regardless of travel destination) was observed for the period from 1 October to 30 April in 2018 vs 2019.

During the same period in 2018, 4,698 patients were tested for CHIKV infection of which, 50 (1.1%) were identified as probable and confirmed CHIKV cases; only one probable case was known to be acquired in Thailand. One laboratory in Spain diagnosed two CHIKV cases in September 2017 and one in September 2018. As the latter case was a traveller returning from Thailand, sequence information related to this case was included in this study.

## Phylogenetic analysis

During the study period, CHIKV sequences were available for 12 patients, linked to travel to Thailand, in seven laboratories in six countries: nine partial E1 sequences and three full genome sequences. None of the sequences carried the E1-A226V mutation. The partial sequences analysed of the Thailand strains are listed in Table 2.

**Table 2:** Overview of chikungunya virus sequence data provided by EVD-LabNet, 2019

Reference Laboratories	GeneBank accession number	5’-3’ ends of sequenced amplicon^a^	Sample Collection Date	Site of Origin of the imported case (if known)
Austria, Vienna.AUT/02.2019	MN053046	10211–10917	7 February 2019	Unknown
Austria, Vienna.AUT/09.2019	MN053047	10194–10932	25 February 2019	Thailand-Krabi
Austria, Vienna.AUT/09.2019/2	MN053048	10194–10985	25 February 2019	Thailand-Krabi
Austria, Vienna.AUT/12.2019	MN053049	10194–10899	21 March 2019	Thailand-Krabi
Austria, Vienna.AUT/12.2019/2	MN053050	10194–10881	21 March 2019	Thailand-Krabi
Finland, Helsinki-1	MN075149	Full genome	25 February 2019	Thailand-Phuket
Finland, Helsinki-2	MN075150	Full genome	27 February 2019	Thailand-Phuket
Italy (Bologna)	MN047314	9994–11312	2 March 2019	Unknown
Italy (Rome, INMI)	MK986662	10180–10922	4 February 2019	Unknown
Israel (Tel Aviv)	MK992771	10525–11170	17 February 2019	Unknown
Slovenia (Ljubljana)	MK848202	Full genome	21 November 2018	Unknown
Spain (Barcelona)	MN080498	10254–10712	6 September 2018	Unknown

EVD-LabNet: Emerging Viral Diseases-Expert Laboratory Network.

^a^ Reference sequence for determination of 5’-3’ ends of sequenced amplicons: CHIKV S27 – ECSA Africa prototype, GeneBank accession number: AF369024.

Phylogenetic analysis was performed based on E1 partial region 10194–11170 (reference strain CHIKV S27, GenBank AF369024) that was the minimal overlap in all sequences provided. As shown in the Figure, the partial E1 sequences of the strains imported from Thailand clustered together with the CHIKV ECSA-IOL strain that emerged around 2004 resulting in widespread outbreaks. Particularly, they clustered in the sub-group comprising sequences of the recently emerged ECSA-IOL variant (‘Pakistan ECSA-IOL’ variant group, Figure) involved in the 2017 epidemic in Pakistan [15] and Italy [10,17,22]. All European ex-Thailand sequences were highly similar, with nt differences ranging between 0 and 0.006% in the 976 bp overlapping fragment that was analysed.

**Figure:**
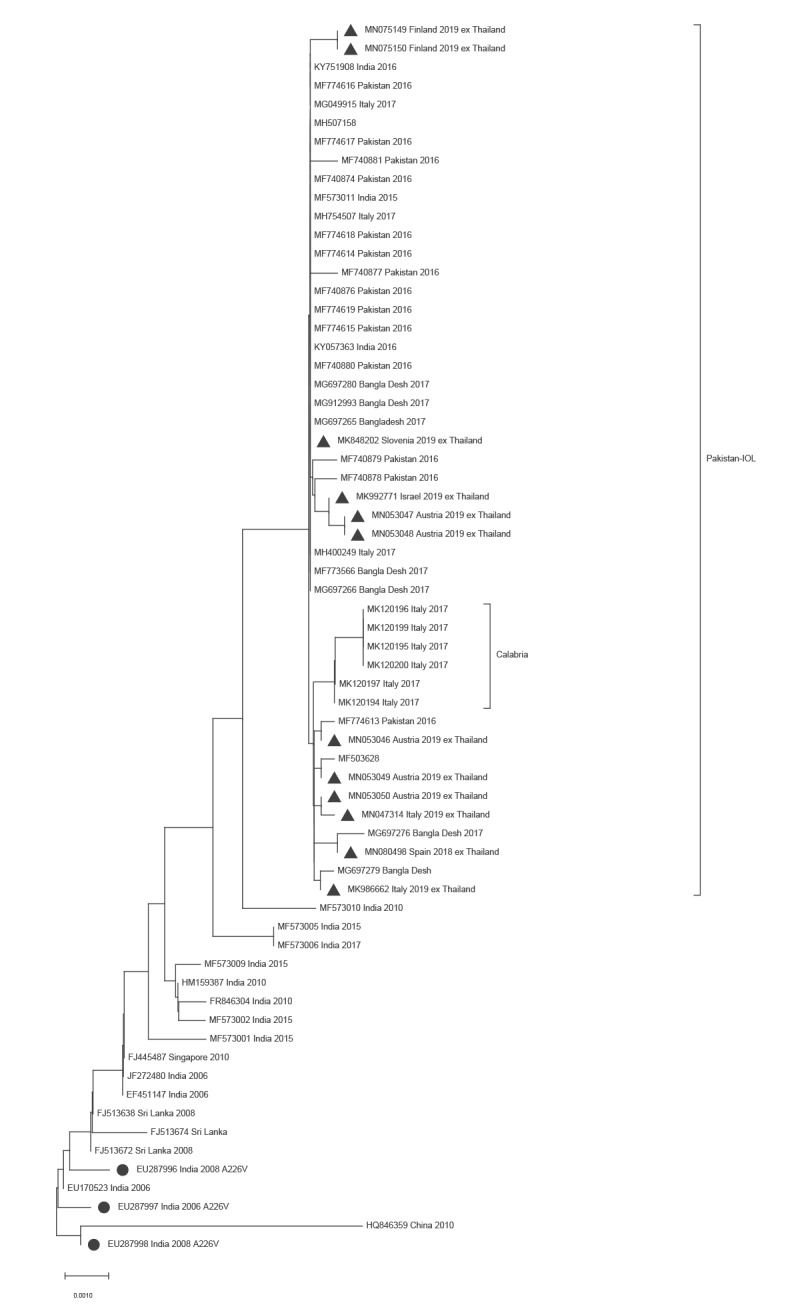
Neighbour-joining phylogenetic analysis of partial E1 region sequences derived from chikungunya virus-positive cases imported from Thailand into Europe, October 2018–April 2019

ECSA-IOL sequences available from the European Nt Archive (ENA) data bank were included in the analysis. Phylogenetic analysis was performed on E1 partial region 10194–11170 (reference strain CHIKV S27, GenBank AF369024) that was the minimal overlap in all sequences provided. Gaps in the alignment due to differences in the length of sequenced amplicons were ignored in the distance model. The phylogenetic tree was constructed using Tamura-Nei model with 1,000 bootstrap reiterations. Two major clusters are highlighted in the Figure: one cluster comprising the ex-Thailand sequences reported here and a second cluster, which includes sequences with the A226V mutation in the envelope E1 gene (indicated with a black circle). The scale bar indicates the percentage of diversity. The branch length of 0.001 was chosen to better show the relatedness of ex-Thai sequences. For each sequence in the tree, GenBank accession number/country of origin of the infection/year of the infection are reported. Sequences characterised in this study are indicated by a black triangle.

## Discussion

In response to the Thailand-CHIKV alert, EVD-LabNet was able to rapidly inventory and share among its members and with ECDC, data on laboratory-diagnosed imported CHIKV cases and sequences. This information directly enhanced risk assessment at ECDC by informing experts during the daily round table meeting where active communicable disease threats are reviewed. While CHIKV is classified as a notifiable disease in the EU [23], reporting of pathogens like CHIKV to ECDC through the European surveillance system (TESSy) does not happen on a real-time basis with regard to returning travellers. TESSy data are usually reported in annual epidemiological reports with surveillance atlas data typically published ca 1 year late [24]. Furthermore, regular surveillance systems do mostly not collect data on the total numbers of diagnostic requests that laboratories have received. Such information is, however, relevant for an adequate assessment of the general level of preparedness and awareness among physicians and current pressure on laboratory response through increased diagnostic requests.

The exchange of snapshot laboratory data within a network such as EVD-LabNet has the advantage of a timely provision of essential data and opens the possibility to direct further investigations, for example through phylogenetic characterisation targeting the presence of virulence and/or vector competence markers and validating common diagnostic tests. However, most laboratories processing vast amounts of diagnostic requests indicated that their routine laboratory information management systems are not set up to enable easy extraction of answers to such queries, and some epidemiological parameters, like travel destination, are often not provided. Furthermore, a lack of standardisation across Europe of e.g. protocols (including specific tests used and interpretation), clinical and laboratory case definitions and local situation-driven differences in test requesting behaviour of clinicians will bias data collection. Hence, the potential impact of international laboratory networks as preparedness and response tool can be further enhanced by improvement and standardisation of collection, quality and mining of data.

Nevertheless, we observed a 23.6% increase in CHIKV diagnostic requests across the participating laboratories during the study period. Moreover, we observed an increase in the number of positive laboratory cases, and in particular an increase of CHIKV-infected travellers from Thailand. The place of infection was unknown for many diagnostic requests and confirmed cases, making it likely that the number of requests and cases associated with travel to Thailand might be under reported in this study and highlighting the importance of a complete travel anamnesis not only for diagnostic and clinical purposes but for epidemiological analysis as well [25]. The E1-A266V mutation was not present in the partial CHIKV sequences generated from 12 of 73 imported cases from Thailand (including the Spanish case from September 2018), but the phylogenetic analysis revealed a high similarity with the strain involved in a large outbreak in the Indian sub-continent and the *Ae. albopictus*-sustained outbreak that occurred in Italy in 2017. Based on information from previous CHIKV outbreaks in Thailand, an E1 A226V strain was expected to cause the 2018–2019 outbreak in Thailand [26,27]. The circulation of E1 A226 CHIKV in Thailand highlights the interconnectivity in Asia and the occurrence of outbreak strain replacement in endemic areas.

## Conclusion

The rapid data collection within EVD-LabNet showed that an increased awareness existed among physicians of the possibility of travellers returning from Thailand with an acute CHIKV infection. The timely sharing of the data within the network and with ECDC alerted the network that a further increase in diagnostic requests could be expected while it stressed the need for awareness that with the start of the vector activity season in Europe and the simultaneous upcoming peak in vector activity in the rainy season in Thailand (June–October) increasing numbers of returning viraemic travellers could have been the source for autochthonous CHIKV transmission in areas with competent vector species while the outbreak in Thailand was ongoing. International laboratory strengthening networks like EVD-LabNet have the opportunity to rapidly collect a European overview of imported laboratory cases and associated critical sequence information, and thus have the potential to inform risk assessments of the public health sector with directly relevant information thereby filling an important role in strengthening preparedness and response.

## CHIKV Working Group

Claudio Argentini, Christopher Barbara, Concetta Castilletti, Pascal Cherpillod, Christian Drosten, Niklas Edner, Philip Eisermann, Olivier Engler, Sandra Essbauer, Claudia Fortuna, Guillaume Fournier, Paolo Gaibani, Valeria Ghisetti, Roger Hewson, Lyudmila Karan, Gulay Korukluoglu, Miša Korva, Isabelle Leparc-Goffart, Giulia Marsili, Aleksander Michalski, Maria Grazia Milia, Richard Molenkamp, Jessica Navero-Castillejos, Laetitia Ninove,Irene Pedrosa-Corral, Maria Carla Re, Sara Sanbonmatsu-Gámez,Teemu Smura, Jelena Storozenko, Olli Vapalahti, Caterina Vocale, Alexander Zoufaly
